# Insights on electronic cigarette products from reviews on the Reddit forum

**DOI:** 10.18332/tpc/111533

**Published:** 2019-09-12

**Authors:** Jon-Patrick Allem, Anuja Majmundar, Likhit Dharmapuri, Jennifer B. Unger, Tess Boley Cruz

**Affiliations:** 1 Keck School of Medicine, University of Southern California, Los Angeles, United States; 2 Department of Computer Science, University of Southern California, Los Angeles, United States

**Keywords:** e-cigarette, vaping, pod mod, Reddit, product reviews, appeal

## Abstract

**INTRODUCTION:**

E-cigarette devices and their component parts are continuously evolving. Little is known about the product design features that may increase the appeal of e-cigarette use, ultimately affecting continuation of use. Product reviews have been described as useful in helping to forecast the popularity of products, and online reviews have become an important channel of product information. This study analyzed e-cigarette product reviews to attempt to arrive at a greater understanding of the features of e-cigarette products that may make them appealing to current users.

**METHODS:**

Data included 248 product reviews found on a popular e-cigarette-related discussion site of Reddit from 10 April 2017 to 12 November 2018. For this study, we examined the sub-sections relating to the positive features (PFs) and negative features (NFs) of the product, found in each review. Common themes were identified and coded.

**RESULTS:**

There were 2929 comments on PFs (average 12 per product), and 1003 on NFs (average 4 per product). Commonly found in the reviews were the ten themes: build quality, color, tip model, battery quality, price, screw quality, power mode performance, coil performance, temperature control performance, and tank quality.

**CONCLUSIONS:**

Findings suggest e-cigarette users expect well-made devices and have developed ways to discriminate between products that perform well from those that do not. Findings suggest that the control of price and design features of e-cigarette products (e.g. color, size, voltage, wattage, coils) warrants consideration for future research and policies addressing e-cigarette use.

## INTRODUCTION

After decades of decline in the use of combustible tobacco products such as cigarettes, new tobacco products such as electronic cigarettes (e-cigarettes) are becoming increasingly popular among adults and youth in the United States and worldwide^1-5^. Some e-cigarette products might be less harmful than combustible products, but there is still risk to health from the nicotine, the specific flavorings and compounds produced by the aerosolization (vaporization) process^6^. E-cigarettes may contribute to initiation of cigarettes among adolescents^7^, and/ or recruit youth and non-smokers to nicotine experimentation and dependence. The effectiveness of e-cigarettes for cigarette smoking cessation is still unknown^8,9^. However, recent research suggests that e-cigarettes are more effective for smoking cessation than nicotine-replacement therapy, when both products are accompanied by behavioral support^10^.

Product design features elicit lasting psychological and behavioral responses among consumers^11^. Currently, e-cigarette products vary in their construction, appearance, composition of e-liquids, and component parts (e.g. cartridge capacity, coil type, heating mechanisms, and battery size)^12^, which may have implications for product appeal, frequency of use, and associated health effects^13^. For example, open-system pod mods, offering flexibility of use in terms of nicotine and cannabis vaping, raise concerns about abuse liability^14^. Devices with variable wattage, also often release high levels of harmful toxicants under higher power conditions^15^. Replaceable coils in devices produce dense vapor^16^, which is an appeal factor communicated on social media in the form of ‘cloud chasing’ (blowing large clouds)^17^. E-cigarette users generally transition between device types, using modifiable devices (rather than first generation or ‘cigalike’ products) more regularly as a function of time and as their familiarity grows with the products^18,19^. Further information on product design features may prove helpful in understanding the growth in popularity of e-cigarettes and changes in patterns of use over time.

Publicly accessible data from social media platforms (like Twitter and Instagram) have been used in prior research to capture and document the environmental context and appeal of e-cigarette use^20-22^. Such platforms may also be able to provide insight to the product design features of e-cigarettes that increase their appeal to current and potential users. Social media platforms facilitate rapid data collection, capturing organic conversations about topics of great import (like e-cigarettes) from the public in their own words. In this way, information pulled from social media can be treated like a focus group^20-22^.

Reddit (reddit.com) is a popular online discussion forum that allows users to create, vote, and comment on posts covering a wide range of topics called sub-reddits. In January 2019, Reddit was estimated to be the 5th most popular website in the United States and 18th most popular website in the world^23^. Exposure to online reviews of products, a form of electronic word-of-mouth^24^, is associated with high purchase intentions^25^ and facilitates decision making^26^. Product reviews have been described as useful in helping to forecast the popularity of products^27^. In some instances, sub-reddits contain product reviews as part of their ‘wiki’ (a webpage that allows collaborative editing of its content and structure by its users). Reddit has been used in prior research focused on tobacco use^28-30^, cannabis use^31-32^, and other health-related areas^33-35^. Reddit is a platform where consumers may collaborate to seek information, share questions and experiences of products^36^. To date, prior research has not examined e-cigarette product reviews on Reddit As such, this study analyzed e-cigarette product reviews on Reddit to attempt to arrive at a greater understanding of the features of e-cigarette products that may make them appealing to current users.

## METHODS

Data collection comprised a sample of product reviews found on the popular (>166 thousand subscribers at the time of data collection) e-cigarette-related sub-reddit (https://www.reddit.com/r/electronic_cigarette/). A sub-reddit can be considered an online community that discuses a specific topic, which is governed by its own set of rules that are enforced by its active subscribers and moderators. The product reviews were ‘scraped’ (copied using a computer program written in Python)^37^, from the sub-reddit’s ‘wiki’ (https://www.reddit.com/r/electronic_cigarette/wiki/anthony_vapes_reviews) - a community-maintained set of documents containing, in addition to product reviews, vaping tutorials, responses to frequently asked questions to aid e-cigarette users and guide new subscribers to the sub-reddit.

At the time of data collection (15 November 2018), there were 248 reviews in total, each for a different product. Reviews were dated from 10 April 2017 to 12 November 2018. Each review consisted of several sub-sections, some exclusive to a single product, while others were common among all products. For this study we examined the sub-sections relating to the positive features (PFs) and negative features (NFs) of the product found in each review. There were 2929 comments on PFs (average 12 per product), and 1003 on NFs (average 4 per product).

To systematically identify commonly occurring themes in the PF and NF sections of the reviews, the data (e.g. the entire text from each specific review) were pre-processed by: 1) lower casing all words, 2) removing stop words (e.g. words such as ‘a’ and ‘the’) and punctuations, and 3) lemmatizing words (e.g. breaking down words into their basic form by removing inflections and variants). Two authors read the most commonly occurring set of words and phrases in the reviews. From this initial assessment of the data, ten themes were identified by the authors. Most PF and NF comments were four to five words long, and could be classified into one (or more) of ten themes (Table 1) with simple word or phrase matching. Phrase matching is accomplished by looking for the presence of all words in a document where the order of words does not matter. The words used to identify themes are provided in Table 1. Themes are presented as percentages in reviews. Multiple themes could occur in each PF and NF section of a review. To illustrate findings, we paraphrased sections of reviews for themes.

**Table 1:** Ten themes (in bold), and common words used to classify a theme as a positive feature (PF) or negative feature (NF) in the e-cigarette product reviews examined (N=248)

*Theme and common words*	*Positive feature (PF) % (n)*	*Negative feature (NF) % (n)*
**Build quality** build and quality	77.82 (193)	4.44 (11)
**Color** color colors bright blue green amber	74.60 (185)	13.71 (34)
**Tip model** tip pin standard 510 810 mm proprietary	58.87 (146)	13.71 (34)
**Battery quality** battery batteries life good compatibility decent size damage small large big door	42.34 (105)	23.79 (59)
**Price** price	40.73 (101)	3.63 (9)
**Screw quality** screw and quality	24.60 (61)	1.61 (4)
**Power mode performance** watt and mode power and mode	20.97 (52)	5.65 (14)
**Coil performance** coil	9.68 (24)	9.68 (24)
**Temperature control performance** ‘temp’ and ‘control’, ‘tc’	4.84 (12)	11.69 (29)
**Tank quality** Tank and quality	4.84 (12)	0

Data collection and analyses were approved by the Institutional Review Board of the authors’ university.

## RESULTS

### Build quality

Build quality appeared as a PF in 77.82% (193/248), and as an NF in 4.44% (11/248), of all reviews (Table 1). When discussing build quality as a PF, reviews often mentioned good quality threading, an excellent tank, and the absence of the sound of a button rattle for a sturdy device. While NFs often mentioned were that the build quality was disappointing, cheap, and parts rattled when used.

### Color

Color appeared as a PF in 74.60% (185/248), and as an NF in 13.71% (34/248), of all reviews. When discussing color as a PF, reviews often mentioned the variety in color options, brightness of colors, ability to match color across component parts, and the finish of the product. When discussing color as an NF, reviews often mentioned the lack of color options (color was only available in black) and if component parts did not match in color.

### Tip model

The tip model (the mouthpiece attached to an e-cigarette device as an alternative to cartridges and cartomizers) appeared as a PF in 58.87% (146/248), and as an NF in 13.71% (34/248), of all reviews. When discussing tip model as a PF, reviews often mentioned that a purchase came with multiple tips, were standard in size, and of good quality. While NFs often mentioned were loose fitting tips, tip shortness, that a purchase came with only one tip, and that the tip was proprietary, or not the standard industry size.

### Battery quality

Battery quality appeared as a PF in 42.34% (105/248), and as an NF in 23.79% (59/248), of all reviews. When discussing battery quality as a PF, reviews often mentioned long battery life, ease with which the battery could be accessed or replaced, small size, and compatibility across products. When discussing battery quality as an NF, reviews often mentioned the lack of a battery life indicator or meter, as well as difficulty in opening the battery door.

### Screw quality

Screw quality (e.g. responsible for holding parts together, usually the tank to the battery) appeared as a PF in 24.60% (61/248), and as an NF in 1.61% (4/248), of all reviews. The quality of the screws was described as good in PFs, or of poor quality in NFs.

### Power mode performance

Power mode performance (e.g. wattage) appeared as a PF in 20.97% (52/248), and as an NF in 5.65% (14/248), of all reviews. Good power or lacking power distinguished PFs and NFs.

### Coil performance

Coil performance (e.g. rating, life, and resistance of the heating element) appeared as a PF in 9.68% (24/248), and as an NF in 9.68% (24/248), of all reviews. When discussing coil performance as a PF, reviews often mentioned compatibility across products, good flavor, lifespan, and a purchase coming with multiple clearly labeled coils. When discussing coil performance as an NF, reviews often mentioned poor lifespan and performance, as well as lack of options and appropriate labels.

### Temperature control performance

Temperature control performance appeared as a PF in 4.84% (12/248), and as an NF in 11.69% (29/248), of all reviews.

### Tank quality

Tank quality (e.g. responsible for holding juice) appeared as a PF in 4.84% (12/248) of all reviews. Similar to screw quality as well as temperature control performance, the tank quality was described as good or bad in PFs or NFs, respectively.

### Price

Price was mentioned as a PF in 40.73% (101/248), and as an NF in 3.63% (9/248) of all reviews. Products priced in the range 30–39 US$ were most commonly mentioned as a PF, while products priced >50 US$ were most commonly mentioned as an NF (Table 2).

**Table 2:** Frequency of price mentions in all e-cigarette product reviews examined (N=248)

*Price (US$)*	*Number of mentions as positive feature (PF)*	*Number of mentions as negative feature (NF)*
<10	NA	NA
10–19	2	1
20–29	22	NA
30–39	33	NA
40–49	6	NA
≥50	6	2

## DISCUSSION

The themes identified in this study of e-cigarette product reviews on Reddit provide insight to the device features that may appeal to e-cigarette users. Findings suggest that e-cigarette users expect quality devices, and have developed ways to discriminate between devices that perform well from those that do not. These include good quality threading, an excellent tank, and lack of rattling or loose component parts. Easy access to the battery with a well-functioning battery door, sturdy tips, screws, and component parts that are compatible across different products were also noted as positive features of e-cigarette devices. Members of tobacco-related forums may represent a ‘hobbyist culture’ that actively engages in information exchange, including discussions of equipment and techniques^38^. Our findings echo this position. Researchers in tobacco control may want to further explore discussion forums, like Reddit, to better understand what drives the appeal of tobacco products, to inform regulation.

Many themes, identified in this study, point to the importance of performance of e-cigarette device components, including the coil, tip, tank, and other parts. Prior research has reported that e-cigarette users prefer advanced devices to the initial lines of e-cigarette or ‘cigalike’ products^39^. Earlier research has also reported that experienced e-cigarette users prefer devices that are capable of being modified with variable voltage and/or wattage for a specific experience such as the ability to create a thick/milky cloud^40^. This poses a public health concern as devices with variable wattage often release high levels of harmful toxicants under high power conditions^15^.

Color was identified as a prominent theme in the present study. Findings suggest that e-cigarette users approve of devices offered in a variety of colors, especially bright colors. Recent work suggests that product appearance, including device color, is a predominant theme among discussions about e-cigarettes on Instagram^14^. Emphasizing product appeal by highlighting device color has also been a key e-cigarette marketing strategy^41^. Device appearance, e.g. standardizing color, may be one of many features that could be considered a target in future policies addressing e-cigarette use.

Price was also identified as a prominent theme. The findings suggest that e-cigarette users find it reasonable to pay 30–39 US$ for their devices. Earlier focus groups among adolescents and young adults demonstrated that high costs of e-cigarette devices motivated discontinuation among lifetime e-cigarette users, while low cost was identified as a reason for e-cigarette experimentation^42^. Future targets for regulation could involve taxation of e-cigarettes to address concerns related to experimentation and abuse liability.

Compatibility across multiple products, and size were common aspects of the battery quality theme. Small battery size was identified as preferable, possibly considered sleeker, pointing to another design feature that could be regulated in the future. Prior research has documented that the overall size and sleek shape of e-cigarette devices (especially JUUL) were discussed features on social media including among youth^22,43^. Modular and compatible e-cigarette designs may facilitate transition across different types of devices (e.g. closed-system, open-system, mods, pods) and raise implications for abuse liability. Future targets for regulation could involve standardizing the shape of e-cigarette devices (e.g. making them larger and ultimately more awkward to conceal), and limiting their compatibility (e.g. closed-systems).

### Limitations

The present study focused on reviews found on Reddit, and findings may not be generalizable to other social media platforms. The reviews analyzed in this study were collected for a 19-month period, and may not be applicable to other time periods. Details about the source of each review were not provided on Reddit. Therefore, these reviews may not reflect all current e-cigarette users’ opinions about each product. However, each sub-reddit is community-maintained, suggesting some degree of community agreement over the content of reviews. This study could not determine the impact of these product reviews on consumer behavior, but product reviews have become an important channel of product information^44^, and have been described as useful in helping to forecast the popularity of products^27^.

## CONCLUSIONS

Build quality, device component quality (screw, coil, battery, tank), performance, color, price and size were common features discussed in reviews on e-cigarette products. Controlling the design features of e-cigarette products, for example standardizing size, color and wattage, to make them appeal to adult cigarette smokers attempting to quit, but not to youth or non-smokers, warrants consideration for future research and policies addressing e-cigarette use.
